# Facilitators and Barriers to Sustainable Employment After Spinal Cord Injury or Acquired Brain Injury: The Person's Perspective

**DOI:** 10.3389/fresc.2022.872782

**Published:** 2022-07-08

**Authors:** Katarzyna Karcz, Barbara Schiffmann, Urban Schwegler, Stefan Staubli, Monika E. Finger

**Affiliations:** ^1^Swiss Paraplegic Research, Nottwil, Switzerland; ^2^Department of Health Sciences and Medicine, University of Lucerne, Lucerne, Switzerland; ^3^Swiss Paraplegic Centre, Nottwil, Switzerland

**Keywords:** sustainable employment, vocational integration, qualitative study, spinal cord injury, acquired brain injury, affected person's perspective

## Abstract

**Background:**

Sustaining employment after initial return to work represents a major challenge for people with a disability. While individuals with spinal cord injury (SCI) and acquired brain injury (ABI) make a prime example for this challenge, their view on factors supporting and hindering sustainable employment have rarely been investigated in depth so far.

**Purpose:**

To examine facilitators and barriers to sustainable employment, as perceived by persons with SCI or ABI.

**Methods:**

Fourteen focus groups and four individual interviews were conducted and thematically analyzed.

**Results:**

Perceived facilitators and barriers to sustainable employment reflected the three biopsychosocial areas of personal, impairment-related and environmental factors. For both condition groups, key facilitators included environmental factors (i.e., aspects of the work organization, the workplace, supportive private and work environment) and personal factors (i.e., the ability to self-advocate, to communicate and to learn how to live with one's own disability). Major barriers comprised injury-related impairments, including decreased mobility and pain for people with SCI and fatigue and limited cognitive resources for persons with ABI, as well as environmental factors related to insurance procedures and the social security system for both conditions.

**Conclusions:**

The biopsychosocial factors identified in our study as well as their interplay should receive particular attention to optimally support sustainable employment in vocational integration and work retention practice. Interventions should particularly focus on the empowerment of those affected as well as on the creation of supportive work environments that match their abilities and needs.

## Introduction

Sustaining work reflects a major challenge for people with a disability, as evidenced by premature withdrawals from the labor market and a significantly decreasing employment rate over the life course (1–3). While return to work (RTW) is a primary goal of vocational integration services, many affected persons face problems in maintaining their jobs after initial RTW and tend to drop out from the labor market in the long run (3–7). By contrast to the predictors of a successful RTW, factors that determine labor market dropouts and a sustainable employment over time are hardly investigated in the literature.

Sustainable employment is defined as “a person–job–workplace match that enables a person to stay healthy and satisfied at work over time, with a work performance that meets the expectations of the person and the employer” (8). Ensuring such a sustainable work situation by creating best possible conditions for a stable employment is a key goal of vocational integration services. While it has been shown that work is generally positively associated with health, well-being and life expectancy, work can also negatively affect physical and mental health as well as well-being if work conditions are unfavorable (9, 10).

For individuals with spinal cord injury (SCI) and persons with acquired brain injury (ABI), achieving a sustainable employment after RTW is particularly challenging (6, 7, 11). While SCI impairs the spine and ABI the brain, both conditions strongly limit functional abilities that are critical in today's labor market: mobility, cognitive functioning, work and social life (12–14). SCI and ABI are conditions with partly different (e.g., physical vs. cognitive impairments, different secondary health complications) but also overlapping consequences (e.g., reduced mobility and flexibility, emotional problems). Together, these consequences comprehensively reflect most of the challenges faced by people with neurological conditions (12–14). Additionally, up to 39% persons with SCI sustain also an ABI (15). A complementary look at these two conditions may not only enable a better understanding of condition-specific facilitators and barriers to sustainable employment but also those for other common neurological conditions (e.g., multiple sclerosis). Based on this insight, general recommendations for vocational integration and job retention practice for neurological disorders may be derived.

In Switzerland, a wide range of curative, rehabilitative and integration services are provided to support health, work and community participation of persons with disability including people with SCI and ABI (16). These services are either financed by the mandatory health insurance, by accident insurance providers such as the Swiss Accident Insurance (Suva) or by the Swiss Disability Insurance (IV). The IV in particular, but in an early stage of rehabilitation also accident insurances, fund vocational rehabilitation and integration services that aim to return individuals to the labor market. However, because the Swiss health care and social security system is complex, fragmented and has a poor case coordination, both the type and the timing of integration services received may differ. The amount of disability pension a claimant is entitled to depends on whether the injury was caused by an accident or an illness, since accident insurances, unlike health insurances, pay a supplementary pension to the IV. Although Switzerland ratified the UN convention on the rights of persons with disabilities (17), there are currently no laws that force employers to employ people with a disability. Job seekers with a disability are therefore exposed to the open, competitive labor market.

There are currently around 6,000 people with a traumatic or non-traumatic SCI and 130,000 people with a traumatic brain injury or stroke living in Switzerland, the majority of them being of working age (18, 19). The employment rate of people with SCI is with 61% about 20% points lower compared to the general population (20, 21). Statistical data on the employment situation of persons with ABI living in Switzerland is currently lacking but, as previously shown, sustaining work after the onset of an SCI or ABI represents a major challenge for those affected (6, 7, 11). In addition, Swiss patient organizations for people with SCI and ABI report a substantial number of enquiries from individuals who face problems in maintaining their jobs.

Two recent scoping studies conducted by the authors of the present article (6, 7) show that evidence on factors that influence sustainable long-term employment of persons with SCI or ABI is still scarce, both internationally and for Switzerland. In-depth and contextualized evidence on factors that facilitate or hinder sustainable work is needed to improve support services that help to prevent early exits from the labor market of those affected. Ideally, generating such evidence should comprehensively incorporate the perspectives of different stakeholder groups involved in the vocational integration and job retention process, with a special focus on the experiences of those affected as well as their employers^1^ and health professionals^2^. The application of a qualitative approach to study the experiences of those affected enables the collection of first-hand information on the dynamic interplay between factors influencing sustainable work that is key to design adequate strategies to prevent labor market dropouts. The objective of this study was thus to identify factors influencing sustainable employment, as experienced by people with SCI or ABI. More specifically we aimed to: (1) detect perceived facilitators and barriers to sustainable employment, (2) examine their perceived relevance, and (3) investigate similarities and differences in the perceptions of people with SCI and ABI.

## Methods

To address our research question, we conducted focus groups (22, 23) and semi-structured interviews (22, 23) with people with SCI or ABI and analyzed them thematically (24, 25).

### Recruitment Strategy and Sample

We recruited persons with SCI or ABI who had achieved a stable work situation after initial RTW, i.e., individuals who worked either full or part time for at least 2 years after the onset of their injury. To gain a broad overview of facilitators and barriers to sustainable employment, we aimed to recruit persons of different age groups and employment status (i.e., employed and unemployed) at time of the study. The inclusion of currently unemployed (but formerly employed) people and of retired individuals allowed us to collect information on factors contributing to labor market dropouts and sustainable work over the life span. Three different recruitment strategies were applied: (1) publication of study announcements in the magazines and on the websites of the national organizations for people with SCI (Swiss Paraplegic Association) and ABI (Fragile Swiss), where we asked interested persons to contact us; (2) direct invitation letters to preselected participants of the Swiss Spinal Cord Injury Cohort study (SwiSCI), representing the national cohort study on people with SCI living in Switzerland (26); and (3) distribution of study flyers *via* vocational integration and health professionals as well as contact persons from the Swiss Paraplegic Association and Fragile Swiss who informed us about the contact details of persons who were interested to participate.

Ethical approval for our research was obtained from the ethics committee of North-West and Central Switzerland (EKNZ, study reference 2018-01317). At the first contact, we informed candidate participants about the study and if they fulfilled the inclusion criteria, they were invited for a focus group discussion. Written consent was obtained from each participant during the focus group. To enrich our data, we offered individual interviews to participants who indicated that they were unable to participate in a focus group. This allowed us to also collect the experiences of participants with higher injury severity (e.g., complete tetraplegia), severe impairments (e.g., aphasia) and challenging private situations (e.g., single mother of two young children).

We conducted a total of 14 focus groups (seven for each health condition) and four semi-structured individual interviews. Overall, 29 persons with SCI and 22 persons with ABI participated in the study, with the majority of them (i.e., 40 out of 52 participants) being engaged in paid employment at the time of the interviews. We scheduled focus groups with three to five participants. To enable persons with attention deficits or higher fatiguability an active participation throughout the focus group discussion. Sample characteristics are presented in Tables 1, 2.

**Table 1:** Characteristics of study participants with SCI.

	**Age (years)**	**Injury type**	**Gender**	**Education**	**Employment status**	**Pre–injury job title (assigned to ISCO−08 major groups)**	**Current/last job title (assigned to ISCO−08 major groups)**	Weekly workload (%)^*^	**Time since injury (years)**
1	30–39	Tetraplegia	Female	University or college	Employed	In education at the time of injury	Clerical support workers	60	10–19
2	30–39	Tetraplegia	Male	University or college	Employed	Technicians and associate professionals	Managers	50	10–19
3	30–39	Paraplegia	Male	University or college	Unemployed	In education at the time of injury	Service and sales workers	0	10–19
4	30–39	Tetraplegia	Female	University or college	Employed	Injury in childhood	Professionals	40	20–29
5	30–39	Paraplegia	Male	Vocational and professional training	Unemployed	Plant and machine operators, and assemblers	Service and sales workers	0	<10
6	30–39	Tetraplegia	Female	University or college	Employed	In education at the time of injury	Service and sales workers	Self-employed	20–29
7	40–49	Tetraplegia	Female	University or college	Employed	In education at the time of injury	Professionals	Missing	20–29
8	40–49	Paraplegia	Female	University or college	Employed	In education at the time of injury	Professionals	80	20–29
9	40–49	Paraplegia	Male	University or college	Employed	Professionals	Professionals	70	10–19
10	40–49	Paraplegia	Female	University or college	Employed	In education at the time of injury	Professionals	40	20–29
11	40–49	Paraplegia	Male	Missing	Unemployed	Technicians and associate professionals	Clerical support workers	0	20–29
12	40–49	Tetraplegia	Male	Vocational and professional training	Employed	Missing	Professionals	80	20–29
13	40–49	Paraplegia	Male	University or college	Employed	Professionals	Professionals	50	20–29
14	40–49	Paraplegia	Female	Vocational and professional training	Employed	Clerical support workers	Clerical support workers	30	10–19
15	40–49	Tetraplegia	Male	Vocational and professional training	Employed	Professionals In education at the time of injury	Clerical support workers	60	30–39
16	40–49	Paraplegia	Male	University or college	Unemployed	Professionals	Professionals	0	10–19
17	40–49	Paraplegia	Female	Vocational and professional training	Unemployed	Injury in childhood	Clerical support workers	0	40–49
18	50–59	Paraplegia	Female	Post-graduate	Employed	In education at the time of injury	Professionals	Missing	20–29
19	50–59	Paraplegia	Male	Vocational and professional training	Employed	Craft and related trades workers	Technicians and associate professionals	50	10–19
20	50–59	Paraplegia	Male	Vocational and professional training	Employed	In education at the time of injury	Clerical support workers	50	10–19
21	50–59	Tetraplegia	Female	University or college	Employed	In education at the time of injury	Professionals	80	40–49
22	50–59	Paraplegia	Male	Vocational and professional training	Unemployed	Craft and related trades workers	Managers	0	10–19
23	60+	Paraplegia	Male	Vocational and professional training	Unemployed	In education at the time of injury	–	0	40–49
24	60+	Paraplegia	Male	Vocational and professional training	Employed	Technicians and associate professionals	Craft and related trades workers	30	40–49
25	60+	Paraplegia	Male	University or college	Employed	In education at the time of injury	Technicians and associate professionals	50	10–19
26	60+	Paraplegia	Male	Vocational and professional training	Employed	Clerical support workers	Professionals	70	40–49
27	60+	Tetraplegia	Male	Vocational and professional training	Employed	Clerical support workers	Professionals	Self-employed	40–49
28	60+	Tetraplegia	Male	Vocational and professional training	Employed	Clerical support workers	Professionals	60	40–49
29	60+	Paraplegia	Male	University or college	Retired	In education at the time of injury	Clerical support workers	0	40–49

* *ISCO-08, International Standard Classification of Occupations by the International Labor Organization (ILO) (29)*.

^*^Full weekly workload in Switzerland is equal to 42 h per week.

**Table 2:** Characteristics of study participants with ABI.

	**Age (years)**	**Gender**	**Education**	**Employment status**	**Pre-injury job title (assigned to ISCO−08 major groups)**	**Current/last job title (assigned to ISCO−08 major groups)**	Weekly workload (%)^*^	**Time since injury (years)**
1	30–39	Male	Secondary education	Employed	Technicians and associate professionals	Clerical support workers	80	10–19
2	30–39	Male	University and college	Employed	Manager	Clerical support workers	15	<10
3	30–39	Female	University and college	Employed	Professionals	Professionals	80	<10
4	30–39	Male	Secondary education	Employed	Manager	Service and sales workers	70	<10
5	30–39	Female	Secondary education	Employed	Professionals	Professionals	50	<10
6	30–39	Male	University and college	Employed	Professionals	Professionals	Self-employed	10–19
7	40–49	Female	University and college	Employed	In education at the time of injury	Clerical support workers	Missing	10–19
8	40–49	Male	Secondary education	Unemployed	Professionals	Professionals	0	10–19
9	40–49	Female	University and college	Employed	Service and sales workers	Service and sales workers	10	10–19
10	40–49	Female	University and college	Employed	Missing	Clerical support workers	50	<10
11	40–49	Female	Secondary education	Employed	Professionals	Technicians and associate professionals	40	20–29
12	40–49	Female	Primary education	Unemployed	Craft and related trades workers	Agricultural worker	0	10–19
13	40–49	Female	University and college	Employed	Clerical support workers	Clerical support workers	30	10–19
14	40–49	Male	University and college	Employed	Technicians and associate professionals	Technicians and associate professionals	50	<10
15	50–59	Male	Primary education	Employed	Craft and related trades workers	Craft and related trades workers	Self-employed	20–29
16	50–59	Male	University and college	Employed	Missing	Service and sales workers	40	10–19
17	50–59	Female	Secondary education	Retired	Service and sales workers	Service and sales workers	0	<10
18	50–59	Female	Primary education	Employed	Professionals	Professionals	50	<10
19	50–59	Female	Vocational and professional training	Unemployed	Professionals	Service and sales workers	0	20–29
20	50–59	Female	University and college	Employed	Managers	Managers	80	10–19
21	50–59	Male	Secondary education	Employed	Clerical support workers	Clerical support workers	20	20–29
22	60+	Male	University and college	Employed	Technicians and associate professionals	Professionals	20	10–19

* *ISCO-08, International Standard Classification of Occupations by the International Labor Organization (ILO) (29)*.

^*^Full weekly workload in Switzerland is equal to 42 h per week.

### Data Collection

The focus group discussions lasted between 90 and 120 mins and took place in seminar rooms that were located in large cities of Northern, Eastern and Central Switzerland. Individual interviews were conducted at participants homes and took between 45 and 90 mins. The first author (KK, a trained qualitative researcher with a background in psychology and sociology) participated in all focus groups as an assistant and note taker. The focus groups and individual interviews followed the same procedure. After a short introduction, the participants were asked to individually write down on post-its factors that helped or hindered them in staying employed. The exact question was: “what helps or hinders you in staying healthy and satisfied at work over time?” After everyone finished, the moderator (MF, a trained physiotherapist experienced in qualitative research) collected the participant notes, read them aloud one by one and requested participants to explain the factors they have listed and the others to comment. The moderator clustered the factors into thematic groups while placing the notes on the pin board to keep them visible for everyone. In the second part of the focus group discussion, the moderator presented factors associated with sustaining work in the long term that were identified in a recent scoping review (6, 7) while asking participants to evaluate the factors' relevance with respect to their own work experience. Participants were then asked to select the three barriers and facilitators they perceived as most important for their own work situation and finally to pick one aspect they would like to improve most when thinking about the sustainability of their own work life. Focus groups and interviews were audio recorded, the records were transcribed verbatim and anonymized.

### Data Analysis

Data were coded by the first author using the software MaxQDA (28). Each factor was first coded separately (e.g., employer support, attitude of co-workers) before factors were grouped into subthemes of similar factors (e.g., employer and colleagues) and themes (e.g., work environment). Finally, subthemes and themes were categorized into three categories (personal, impairment-related and environmental factors), representing three biopsychosocial areas influencing sustainable work. Coding scheme and factor categorization were extensively discussed among the co-authors throughout the coding process. Beside MF, the co-authors included BS (a sociologist experienced in qualitative research) and US (a senior researcher and psychologist experienced in qualitative research). In case of disagreement between the three co-authors who were primarily responsible for the analysis (i.e., KK, MF and BS), US was consulted and factors were discussed until agreement was achieved.

With regard to the prioritization of facilitators and barriers, first we allocated factors that were prioritized by the participants during the focus groups to the corresponding themes. We then checked which themes were found most important within each biopsychosocial area. Finally, we compared these most important themes between the two health condition groups.

To ensure the credibility and validity of the study results, they were presented to and reflected upon with our practice project partners (i.e., Fragile Swiss for ABI; Institute of Vocational Integration (ParaWork) and Outpatient Care Unit at the Swiss Paraplegic Centre for SCI) in three discussion sessions of 90 mins (29).

## Results

Perceived facilitators and barriers to sustainable employment reflected the three biopsychosocial areas of personal, impairment-related and environmental factors. Themes and subthemes of each category are presented in Table 3. The prioritization of facilitators and barriers made by the participants is presented at the end of the results section.

**Table 3:** Overview of identified factors, categorized into themes and subthemes.

	**Facilitators or barriers perceived by participants**	**Exemplary quotations**
**Personal factors**		
Perceived benefits of having a job	Satisfaction, confirmation Being appreciated Being integrated Work provides a structure and purpose	*The work gives you confirmation. You're not just disabled. I can still do something. Everyone is looking for and needs confirmation. So it's something important. And you get that in your job. (SCI_3)* “*Meaningful work” and “enjoyment at work”: Everyone has their nature or an inclination what they like to do, and one should also be efficient. We have a meritocracy. And I think it's very important that people enjoy what we do [at work]. And can effectively make a valuable contribution. (ABI_7)*
Personality characteristics	Positive work attitude Willingness to work Self-advocacy Persistence Resilience Acceptance of changes because of injury Barriers: ambition, self-doubt, putting too much pressure on oneself	*At the beginning I had such barriers in my head. I knew that I was going to study, but I thought, do I even have a chance of finding a job afterwards? But I had a lot of chances afterwards. But hence, my head. In the sense of because it can simply be very beneficial or very hindering. (SCI_6)* *I know both the self-doubt and the unconscious excessive demands. And every time there is self-doubt, that one is not enough. It's an extremely fast society and if you want to keep up, it overwhelms you non-stop. I still have periods like this regularly. When I've overwhelmed myself again, I always think, ah, no. (ABI_4)*
Skills and strategies	Learning to live with a disability Relearning own body and its capabilities (work tasks) Health management Further education/retraining Work experience Communication skills Networking	*I used to keep it [need to go to the toilet] a secret, but not anymore. I say every now and then I have to go to the toilet, if the session isn't over then I have to go. Usually people understand. When I go on excursions with students, I find myself forgetting myself. And then we are somewhere in the middle and then I realize that I have to go. Then I realize that I haven't listened to myself enough. (SCI_6)* *I was extremely pushed to the limit at home and that helped me to understand. And for me it is all about break management: lots of regular breaks. (ABI_4)*
**Impairment-related factors**		
Injury-related impairments and secondary health conditions	(In)visibility of disability/secondary health conditions Injury-related impairments typical for SCI Injury-related impairments typical for ABI	*How stable is your paraplegia? What else do you get because of it? The situation just has to be stable. You know, now I have tetraplegia and I have this and that limitation. If I had pressure injuries and neuropathic pain, things like that had kept bouncing me back, then that would affect my happiness differently. Then you're constantly juggling. (SCI_6)* *Now there are also certain specialist seminars that you can attend, but then I also have the feeling that I couldn't do it at all. Sit in a room for 7 h and just record, that would be just too much, that is no longer possible. (ABI_6)*.
Limitations in work functioning and performance	Lower resilience Lower mobility Unstable health and abilities Lower self-consciousness—risk of overburden and burnout	*I then simply noticed that if I wanted to do something else then work, I couldn't work 100%. It just wasn't physically possible for me. Then at some point this wheelchair thing became a problem, simply the burden. (SCI_2)* *The reduced resilience and quick fatigue. You don't notice me that much: I don't forget right away, I can speak, and yet the quick fatigability. I can no longer process the information immediately and need more energy. (ABI_4)*
Additional challenges related to work resulting from injury-related impairments	Extra time needed due to disability management Extra time needed for rest/recovery Lack of flexibility	*Someone else was promoted even though he wasn't qualified and had less work experience then me. So I looked for a conversation with my boss, who asked: “With the wheelchair, if a call comes in, can you drive to Düsseldorf within 3 h for a conversation on Saturday or Sunday?” I had to say no, I can't. Because you have to plan everything, you're not that flexible. That was the reason I didn't get the job. (SCI_4)* *I found I have two ears, but only one brain, I have to do one thing after the other. Sometimes that's so difficult. I have my to-do list, my schedule, what do I still have to do in the office. I always have to distance myself extremely, send people away. This is exhausting. (ABI_4)*
**Environmental factors**		
Social environment and goodwill of other people	Being understood, accepted Emotional support Support in daily chores, transportation Connections, peer group Financial support (ABI) Help in case coordination/procedures (ABI) Loneliness, exclusion (ABI) A must to explain whole story to every new person (ABI)	*I just think that nowadays you need a lot of connections, use all of networking and tell as many people as possible that you're looking for a job. Yes, lot is about personal relationships. If you know someone there, know someone here, then you just have to get in touch, so that you can build a small network. Because a network also holds you tight. It catches you too. If there are difficulties, then you can always benefit a little from it, because then people will help you too. (SCI_3)* “*No pressure from your partner”: I think that's really great too. So your environment can also put pressure on you when you're annoying. Great understanding of my closest environment, that's one of the most important things. When you're in a wheelchair, everyone sees, ah, he's in a wheelchair. With me you can see it a little, but normally you don't see or notice it. (ABI_7)*
**Work environment**		
Subtheme: Workplace	Barrier-free/adapted workplace/accessibility Possibility to lie down/own office Public transportation/having own car Parking Distance workplace-home Noise, light, open space (ABI)	*The first thing I had to ask was “Would I even be able to get in with an electric wheelchair?” and then I say “No, I can't do that.” And they're definitely not moving because of me. So you can bury it right away. That really is almost the first question. (E_SCI1)* *Noise and light. That's a big issue for me, because it makes me so tired. That's also something I've struggled with a lot. When I was at the Unemployment Office, it was so difficult to explain to them, just to explain to them at the table: “Could I please sit with the window on my back so I don't have to look out the window.” People just look at you with big eyes and then you say: “Sorry, that's just how I work.” (ABI_7)*
Subtheme: Working conditions	Work organization (flexible working hours, home office, being independent, good structure/repeatability, work overload, too long working hours, time pressure) Time management (not) SCI/ABI friendly Lower weekly workload No chances for promotion because of limited mobility Interesting jobs only possible with high weekly workload Work tasks interesting and providing personal growth Holidays, breaks Difficulties to keep a work-family balance	*Toilet, there's one in a six-floor building. There is a disabled toilet on the first floor, then you are with the men. Then you go there and the men say, don't be so stupid, why are you sitting here? This is the time for me to play with my health. I realize I should actually empty the bladder, no, now I'm standing here on the fourth floor and have to go back to the other side, down the elevator. Then I think, no, I'm not going now if I'm playing with my own health. So I have to learn something, but it has to become more natural, all these barrier-free facilities or IV facilities. (SCI_3)* *It's the balance between family and work that makes it so difficult. I'm looking around because I've just noticed that I need a change. It's not really possible, the price is too high to work this 80 percent. At the moment it is mainly or the family or work. And I don't know how much longer I can keep that. (ABI_2)*
Subtheme: Employer and colleagues	Support Lack of knowledge of SCI needs, wrong assumptions Lack of knowledge about ABI and its consequences Low awareness of employer about possible support from IV/SUVA Company values and climate End of rehab perceived by others as becoming healthy again Disregarding post-injury impairment by others	*Many only see the wheelchair. I once had a boss who said: “Yes, you know, my aunt or niece is also in a wheelchair, I can't see the wheelchair anymore. For me, you're an employee like any other.” On the one hand, that's praise, but on the other hand, she just didn't understand what it means to be a tetra with all the other things. (SCI_3)* *A lot of understanding from the team and the boss. On the one hand, when I was reintegrated, I was given the time I needed. The other thing is now, yes, that you can sometimes find me lying on the sofa. That I take more breaks, that sessions are shortened. That your boss also knows a little bit about what it means to have a brain injury. And that you don't look so meticulously at breaks and time, but at work performance. With less working hours with more breaks, I perform better than if I work through. (ABI_4)*
**Services**		
Subtheme: Rehabilitation and reintegration	Bad/good advisory Possibility for workplace adaptations and devices Advisor without SCI/ABI experience Long waiting time for benefits related decisions Long, complicated and unclear administrative procedures	*I was actually lucky, I had contact with a great IV counseling back then, right at the beginning. When I found the spot, we then discussed how long, how much etc. It wasn't someone who just worked through his dossier and didn't even see the person behind it. He really came and took care of it. (SCI_3)* *Every 3 months we had meetings with the insurance where everyone was there: from the employer's point of view, the management and then that of the accident insurance company, which would have actually managed my case, was always so tough and I always had the feeling: she doesn't take me seriously. The IV gave me a lot of support. I have to say that I have had a positive experience with the IV so far. But the pressure was always there. (ABI_3)*
Subtheme: Health professionals	Patient organizations Health assistance	*We paraplegics simply have a little advantage because of ParaWork. They have an agreement with the Disability Insurance and they also take care of reintegration process. And they have a solid medical knowledge about secondary health conditions and so on. I think other forms of disability don't have that. Then some IV advisor comes along who calls himself a coach, but he doesn't know the actual clinical picture. Or like my boss, I see you can write, you're just slower and you're in a wheelchair, but you can't see what's behind it all. (SCI_3)* “*Fragile Suisse.” I always thought, “That's good.” There are people I can talk to. They know that if I can't speak well at the moment, they will wait. I just feel safe there. (ABI_1)*
Subtheme: Insurance	Arguments for benefits/rejecting valid claims Injury related expenses/psych support not covered Bad/good advisory from institutions Fighting for benefits, permanent need for justification, stress Long-term support needed Pressure to perform/work more (especially highlighted by ABI) Importance of having health assistant Complicated procedures/no support/responsibility on ABI person	*The Swiss Paraplegic Association brought to my attention that I was entitled to disability pension, which I never received. I only discovered that three years ago. Then we tried to talk to the insurance, but no information ever came from them. Then I applied, but was rejected. Three times I intervened and three times it was rejected. After 2.5 years I was proved right only because my lawyer persistence. (SCI_5)* *Neither Suva nor IV understood why I still wanted to go to work. I really think that's bad. Just because it is hopeless that I would work more or that they would have to pay less. On the other hand, the good relationship with Suva was very important, they supported me in certain things. But once it came to the job, it was bad, really bad (ABI_7)*
**Systems and policies**		
Subtheme: Social attitudes	Being observed/noticed more than others when absent Need to work more for recognition equal to healthy people Belief that people with a disability would work less Employers unwilling to hire persons with SCI Belittle person's abilities due to disability Pressure to perform and prove own value as an employee Goodwill of others	*I experienced it in my last job when it came to front work, i.e. making a presentation in front of 200 people on an important topic. When it came to my topics, my boss usually took over, so that he presented my topics that I had to prepare and he had no idea about them. I had a very strong feeling that there was something like a protective function, “We'll do it for you”—but in the end he wouldn't have anything to say in terms of technical expertise. (SCI_2)* *Accepting change seems difficult, precisely because you don't see it. When I try to say a name, it doesn't come. Then everyone says “yes, I know that too, I have that too.” That's just not true. It's different and my neuropsychological test confirms. I just have a physical difference and people disregard it. (ABI_3)*
Subtheme: Labor market	Insecurity/fear to lose a job because of rationalization away Digitalization/development as threat for simple jobs Competitive and profit as most important at labor market Pressure to perform	*I have the feeling that you are under special observation. If I miss a day, then: “Uh, is he sick.” But because you're disabled, it's like, “He's not that fit.” You have to prove yourself more. You have to show yourself more and more. They don't expect you to do certain things and you always have to prove yourself more than the others. You have to work twice as well to get the same recognition. (SCI_4)* *It's very difficult to find a job on the job market, because today a workload of 120 is required. When I applied with a lower workload, there and there and there I heard “Yeah no, aha, hmm, oh yeah no, um, yeah we'll get in touch if we have a chance-” and so on. So it is extremely difficult to find anything. And also, to find understanding. And therefore, I am self-employed in a way. (ABI_2)*
Subtheme: Disability and social security policies	Difference because of injury cause: traumatic vs. non-traumatic Fragmentation of support Financial disincentives	*When you get a raise or a promotion, you have to calculate if you really want it. Because if you get 100 francs more and earn in general too much, you lose your social benefits and, in the end, you get 300 francs less. So every promotion you have to think twice about whether it is in your interest or not. That's just bad. This also gives a false spirit. (SCI_7)* *Then there's the financial aspect. I'm alone. I have already said that if I were still married—the father of my children made good money. If I were still with him I could say “I don't have to work.” Or I'm looking for something relaxed. But I have to take care of myself (ABI_3)*.

### Personal Factors

Personal factors comprised the three themes “perceived benefits of having a job,” “personality characteristics” and “skills and strategies.”

#### Perceived Benefits of Having a job

Participants from both condition groups perceived various benefits of having a job, such as social interactions, purpose in life, sense of belonging or providing a structure for everyday life, as facilitators for sustainable employment. They also mentioned that their job gives them satisfaction and recognition, and makes them feel appreciated.

#### Personality Characteristics

With respect to personality characteristics, participants with SCI and ABI reported similar barriers and facilitators to sustainable employment. Facilitators included a positive attitude toward work, persistence and high internal motivation to work or to keep a job, while high self-expectations, ambition and self-doubt reflected barriers to sustainable work.

My head gave me a lot of chances, but at the same time built in a lot of obstacles. Simply because I've always heard from outside prejudices against people with disability. Tolerance toward the outside world is something completely different than toward yourself. I struggle a lot to say: hey woman you have your talents, you have opportunities to move forward. Pack it up! Versus the head that says: Who wants a little cripple like that? Simply the negative spiral, the doubts that can pull you down very deeply. But somehow, I just kept going. Despite all doubts, it went on (SCI_6).

#### Skills and Strategies

Participants with SCI or ABI perceived personal skills such as having a higher formal education (e.g., University degree) and sufficient work experience as facilitators of sustainable employment. The same was true for soft skills like the ability to communicate, to explain one's own needs to others and to self-advocate. Also, self-acceptance and reconciliation with the injury-related limitations in daily living and reduced career opportunities were reported as facilitators. Furthermore, participants described several individual strategies that helped them to stay at work, including the appreciation of little things, sense of humor and openness to others about one's own injury and its consequences.

Another facilitator highlighted by both condition groups was learning to live with a disability, including getting to know medical and legal procedures and becoming aware of one's own rights and capabilities of living and working with a disability. Such knowledge allowed individuals to communicate their needs to the employer, to choose their tasks and workload as well as to align their life with the new needs resulting from their health condition. Becoming an expert in the management of one's own health and the prevention of secondary complications as well as developing a good sense of one's own body and paying attention to its signals was deemed helpful for an adequate energy management to foster stable employment.

I sometimes even notice that I have an infection, purely because of my pain, although I have no idea where the infection is. But I know for sure that I have an infection somewhere and I just notice it through the pain. But I also know that it will pass again, so it is doable (SCI_6).

Low self-awareness appeared to be a particular barrier to sustainable work for people with ABI as low self-consciousness represents one of their invisible post-injury impairments.

The case managers slowed me down, they all warned of the chronic exhaustion: watch out, watch out. I can tell that I'm on the line. I cannot work like that for years. (…) Sometimes it's so difficult. I have my to-do list, my schedule, what else do I have to do in the office, in the administrative areas. I always have to set myself apart extremely, send people away. This is exhausting (ABI_6).

Differences between the conditions became apparent with regard to skills and strategies that were related to particular post-injury impairments and how they influence individuals' educational and vocational opportunities. One strategy that was only mentioned by persons with SCI was to acquire further education or retraining.

The more exclusive you are and the more skills you can bring, the more they will go for it again, because they are looking for someone who can do it. Because professionals in this field are wanted. That's almost the only thing where I can qualify against the healthy people, compensate (SCI_1).

For people with ABI, the strategy of acquiring further education was often not feasible due to the limitations in cognitive abilities and increased fatigability.

### Impairment-Related Factors

Impairment-related factors involved the three themes “injury-related impairments and secondary health conditions,” “limitations in work functioning” and “additional impairment-related challenges at work.”

#### Injury-Related Impairments and Secondary Health Conditions

Both persons with SCI and ABI agreed that injury-related impairments and secondary health conditions are major challenges to their sustainable employment in the sense that dealing with them costs a lot of time and energy. If they are neglected, participants carry a high risk of becoming seriously ill and subsequently being on sick leave. Persons with SCI reported that they have to deal not only with mobility limitations and issues such as urinary and bowel incontinence and sensory loss, but with secondary health conditions such as pain, spasticity, urinary tract infections and pressure ulcers. Persons with ABI mainly reported challenges related to their limitations in cognitive functioning including a lower attention span, concentration and language problems as well as increased fatigue or headaches. Additionally, persons with ABI struggled with the invisibility of the majority of their post-injury impairments. For their co-workers and family, they looked the same as before the injury which may lead to the assumption that they were fully recovered and do not need special support or different treatment anymore. For strangers, they appeared to be healthy people, therefore their sometimes unmatching behavior or facial expression were disturbing. Such situations were considered as stressful by participants with ABI participants and triggered some of them to avoid social interactions and meeting new people.

#### Limitations in Work Functioning

Both condition groups reported a lower work performance due to prolonged time needs for completing work tasks as a major barrier for sustainable work. Participants with ABI perceived themselves as generally slower than other employees and as requiring more work breaks, while participants with SCI expressed that they need more time for moving within and around the building when joining meetings (e.g., extra-time needs for reaching an elevator) or using the toilet (e.g., extra-time needs for toileting due to a poor availability of wheelchair-accessible toilets).

These are things that people around you don't see, but that hinder you accordingly. I have to go to the toilet relatively often and stay there for a relatively long time until my bladder is empty. People I work with know and accept that. It's not a problem at all. But before they always though that I slept on the toilet so I had to explain that too. It takes an awful lot of time (SCI_6).

Both groups of participants reported that they need more time for resting. Persons with SCI reported a prolonged recovery time from illnesses or injuries.

The fact is that I think it is very difficult for someone “normal” to understand that it takes longer until you are really healthy again. It doesn't matter if it's a flu or a broken leg. And it's hard not to get into this spin: “I have to get well again as quickly as possible and perform at my best.” Then everything goes longer because you're not feeling well after all (SCI_1).

An increased need for sleep and for breaks to recharge for being able to work again was perceived as challenging by people with ABI.

It turns out that I can work between 5 and 6 h on average. If I don't take this seriously, I'll break down. But if I work 4 days now, I'll be dead 2 days and have to be totally alone (ABI_2).

#### Additional Impairment-Related Challenges at Work

A main barrier for sustaining work reported by persons with SCI was the additional time they require for managing their health and disability. They often have to organize assistance of professionals or family members to reach their workplace or other appointments or have to invest time for health and disability management that interferes with their working schedules such as therapies, exercises or toileting.

Also, the therapies are not at seven in the evening, as I would like them to be. Rather, in the middle of the afternoon, which is an obstacle for meetings and it's tedious to explain that you have your therapies and can only come at one or three in the afternoon. Then they gave me a funny look (SCI_3).

For persons with ABI an important barrier to sustainable employment was their decreased possibilities for retraining or further education due to their limited cognitive abilities and increased fatigability. In particular individuals with a low weekly workload reported that neither the disability insurance nor their employer was willing to invest in future training because they did not expect a high return on investment.

On the one hand, I am sure that my employer no longer wants to invest too much. Because shortly before my accident I completed my education and what I have learned is already there, but the employer was certainly no longer able to benefit 100% from this. Now there are also certain specialist seminars that you can attend, but then I also have the feeling that I couldn't do it at all. Sit in a room for 7 h and just absorb knowledge, that would be just too much, that is no longer possible. These are things that make you up to date and fit on the job market. For me it is just up to my accident and afterwards I haven't done anything. So, on the one hand it is no longer possible and on the other hand nobody wants to invest any more (ABI_6).

### Environmental Factors

Environmental factors were grouped into four themes: (1) “social environment and goodwill of others,” (2) “work environment” (including the subthemes “workplace,” “working conditions,” and “employer and colleagues),” (3) “services” (covering the subthemes “rehabilitation and integration,” “health professionals” and “insurance”), and (4) “system and policies” (including to the subthemes “social attitudes,” “labor market,” and “disability and social security policies”).

#### Social Environment and Goodwill of Others

Both condition groups perceived the social environment as having considerable impact on the sustainability of their employment. Emotional support from partners, family and friends (e.g., empathy, acceptance or appreciation) was deemed helpful to better manage the everyday challenges of their working life. Private social relationships or having someone to talk in case of work-related problems were seen as a buffer from difficulties experienced at work. Another form of support was financial help or assistance in daily activities. Lack of social support was deemed a major barrier for sustaining employment by both condition groups. Assistance of others was mentioned as indispensable, particularly when organizing child care, holidays or when working outside the office.

My assistants also take care of my children. Without family it's nearly impossible. With Swiss employment contracts, where you can work a maximum of 50 h, I would actually need three assistants. Three full-time employees. Since nobody is allowed to work seven nights, I need two more rooms in my apartment where they could live. It's not possible. So I am dependent on this “human help.” For example, holidays. Then the assistant travels with me voluntarily. I pay for eight and a half hours and she voluntarily gives the rest of it. Otherwise it wouldn't work at all (E_SCI_1).

People with SCI perceived help in transportation and their health routine as critical, while ABI individuals reported that having a support in everyday chores like shopping, cooking or taking care of children allows them to rest after work and to recover for the next working day. People with ABI also mentioned the importance of help in navigating procedures of the social security system and complex formalities that overwhelmed them due to their cognitive limitations.

#### Work Environment

##### Workplace

The proximity of the workplace to one's home represented a supporting factor for both condition groups as a short commute allows them to save time and energy.

For persons with SCI, an adjusted work environment including the accessible work place as well as the adapted work tasks was perceived as a crucial facilitator. While adjustments like ramps, elevators or wheelchair-friendly toilets are already critical in the initial phase after RTW, an own parking space, a wheelchair-friendly building (including automatic doors without doorsteps or easy access to different building parts) and a place to lie down to release pressure from buttocks and spine are important in the long run. Not all participants had access to adequately adapted workplaces and in the long term little physical barriers tended to accumulate and led to frustration.

This goes back to time-management. In the last job we had extremely full calendars and have to move from one meeting to another within 2 mins, preferably on the other side of the building. I always had to look for the lift and was always too late (SCI_1).

Participants with ABI mentioned that working in a multi-space office with several co-workers and intense levels of noise and light distracts them and drains their cognitive resources faster. Having a quiet place to lie down and nap was described as a facilitator for sustainable work.

Participants of both condition groups stated that structural changes in well-functioning workplaces like office or company relocations to different buildings or cities were risk factors for staying employed due to the high adaptation effort required. Moreover, temporary workplaces commonly required a longer commute or were poorly accessible and neither companies nor insurances were willing to invest in temporarily workplace adaptations.

For people with SCI, having a well-adjusted workplace was sometimes a reason for not looking for a different job even in case of low job satisfaction or a wish to change a job. The challenge of finding a job with a well-adjusted workplace or long waiting times for the reconstruction of a future workplace were a barrier to individuals' mobility on the labor market. Several persons mentioned felling a pressure to stay in an already adapted work place, because the IV finance work place adaptations only every several years.

##### Working Conditions

Working conditions were perceived as barriers and as facilitators by both participant groups. Flexible working hours and the possibility for home office were perceived helpful as they allowed individuals to design a working day according to their health-related needs. People with SCI and ABI are both compelled to start their working day later due to extra-time needs for preparing themselves in the morning. Most study participants were thus working part-time, which allowed them to allocate enough time to health management and recovery.

I think it's better to work a little less and then you are fine. Because if you work a little more and then get problems you can't work at all. I basically think that wheelchair users should work a little less so that you can look better for your body. Because that's why you can work longer. If you work 100% at the beginning, after 10 years you have such health problems that you can no longer work at all (SCI_1).

Working part-time or being self-employed allowed individuals to better control their workload and work demands, but it could also constituted a barrier if deadlines accumulated or in the case of last-minute changes in work schedules.

I also experienced the [part-time work] as negative. (…) You get into the situation where you have several part-time jobs. And that's not really possible for me. But I have to. Otherwise I have too little money. (…) I have to handle a 12-h day, depending on the situation. And only in a 20% workload. But it still has to be done. Now I have the burden of the few part-time jobs at the same time (ABI_2).

Participants with SCI perceived working part-time as a reason for not feeling fully integrated into a work team or for communication problems if decisions about work tasks were taken during the person's absence from work. Moreover, part-time work was reported to hamper career progression as most managerial positions require a higher weekly workload. People with ABI experienced a low work percentage as a barrier to ask for workplace adjustments or funding of further education.

Participants of both condition groups considered time pressure at work to be a problematic factor causing faster fatigue and compromising health routine or work-life balance.

(…) and that I can work without time pressure. Because that's exactly what breaks me. When I'm pressed for time, my shop goes down. The problem I have is fatigue. I get tired extremely quickly. I can't take the stress anymore (ABI_6).

##### Employer and Colleagues

A supportive and understanding team and employer were described as key facilitators to stay at work by people with SCI as well as ABI.

I had a very committed colleague before who was taking over some of my responsibilities because she just recognized my situation and saw that something would be difficult for me. When something needed to be arranged and they discussed how to organized it on my absence day, she would stood up for me and said something. I didn't always have to stand up for myself. It takes so much strength. Now, I have colleagues who just don't want to understand my situation. And that's why I sometimes have to work quite long hours, because arrangements are not optimized for me (E_SCI_1).

Poor knowledge of employers and work colleagues regarding the consequences of SCI or ABI were experienced as challenging and led to misunderstandings and exaggerated performance expectations. A lack of knowledge on disability-related adjustment needs at work triggered conflicts and lowered support from employer and colleagues.

Or when an employer doesn't know what you have to do in the morning until you are ready. Or that you don't just lie down because you're so lazy, but that you do that so you don't get pressure ulcers or back problems or shoulder problems or whatever (SCI_1).

Several participants with ABI mentioned that while they generally receive a lot of support and understanding from their employer and colleagues, this support is reduced in stressful situations and in case of time pressure. Additionally, people experienced that co-workers seem to expect from them a full recovery and positive attitude. The fact that this did not happen caused frustration on both sites. While colleagues became tired of the affected persons' problems, participants felt bad because they were unable to meet the co-workers' expectations. Lack of open communication on these issues led to conflicts and decreasing support from colleagues.

M: [answering to the question “how are you”] Just the message from me: Everything is fine again—it never comes. I always say—it depends on whether I'm honest or not—but actually, quite spontaneously: “everything is great,” that [phrase] just doesn't come from me. People are getting tired, they want to hear it so much: everything is fine again.

K: It probably never happens. That's why I always have a poker face (ABI_7).

Finally, changes of the supervisor or in the team were perceived as a risk factor for sustainable employment by both condition groups.

My boss is changing department this summer. I can't imagine getting another manager. Because I also have the feeling that I've been able to set things up as needed now, everyone knows what I've achieved and everything. If a new one comes along, then I can't prove myself (ABI_4).

#### Services

##### Rehabilitation and Integration

Although specifically asked about factors influencing sustainable employment, participants from both conditions often digressed to their RTW phase, indicating the significant influence of this phase on their further employment trajectory. Individuals reported that in the initial phase of RTW they had to learn how to become an expert of their own disability, to understand the consequences of their health condition for daily living and to develop a new identity. Participants from both condition groups indicated insufficient psychological support during the RTW process. Moreover, some participants with ABI mentioned that they missed a dedicated support person who was knowledgeable about their disability and its consequences. Such a person could have helped them to navigate through the complicated rules of the social security system during and after their RTW process. Having talked to participants from many Swiss cantons, we noted considerable regional differences in RTW services and procedures with respect to the engagement and experience of the case managers and the particular rules of the cantonal IV office. People with SCI or ABI both shared positive as well as negative experiences from their RTW phase depending on the support they got and some of them emphasized that this phase was for some of them decisive for their later work life. Participants with ABI would have appreciated a slower pace and less pressure from the insurance company during their RTW period.

The pressure was always there and it was terrible. It seems to me that something should be taken away from this pressure. You make a lot of progress, but you always have this pressure. Then the 2 years are over, they want an expert opinion, interdisciplinary four different doctors. Now they know everything about me, from my childhood and about divorce and everything is questioned. I do so much and I want to be integrated and not just at home. And then there's the financial aspect. I have to take care of myself (ABI_3).

##### Health Professionals

Health professionals, such as family physicians or psychiatrists, and support lines form patient organizations were perceived as key support by people from both conditions. Because a confidential person at the insurance company was often missing, these professionals were often the first contact persons for people with ABI who experienced work-related difficulties. Although family physicians and psychiatrists often lack specialized knowledge on work-related issues, they might be aware of existing support systems (e.g., patient organizations) and can thus guide affected persons accordingly, in particular as not every participant was aware of the support services provided by patient organizations. In particular people with SCI reported that they have strongly benefitted from the well-developed support network of the Swiss Paraplegic Association and the services of the Institute of Vocational Guidance (ParaWork) at the Swiss Paraplegic Centre. For people with ABI, the support system appears to work less effectively, potentially because Fragile Swiss represents a patient organization with less resources to adequately support affected persons.

##### Insurance

Participants from both conditions expressed various positive as well as negative experiences with health, accident and disability insurances, of which some went back to the initial RTW phase. In Switzerland, disability benefits differ depending on whether the injury was caused by an accident or illness, with more favorable benefits for people who suffered an accident. The disability pension level is determined based on individuals' earning capacity at the end of vocational rehabilitation and is calculated based on the difference of their pre-injury earnings and the theoretical earnings they are still able to achieve on the labor market with the disability. This calculation either results in a full, a partial or no disability pension.

Key barriers for sustaining employment expressed by participants with SCI as well as ABI refer to the regular verification-revisions of their disability pension, which typically happen every 5 years, and which are often perceived as stressful events threatening financial security. Participants pointed to time-consuming compiling of documents requested by the IV and the long duration of the revision process.

Pro Infirmis [patient organization] and Fragile gave me legal support because the IV made a big mistake in the calculation. I had no legal idea but people who edited it [the documents], noticed that there is a mistake. And thanks to them I have now received a ¼ pension—after 7 years. Because IV wanted to do a clarification and another one and so on. I have the feeling that those who should help push you the most. (ABI_3)

A second key barrier reported by both condition groups refers to the lack of long-term support services after the end of the vocational integration phase. Due to the lack of an adequate long-term support work-related problems often accumulated and were not addressed until a conflict escalated or the person lost a job. To prevent such situations, participants with SCI and ABI both indicated a strong need for a contact person at the insurance or a vocational integration specialist supporting them in critical situations in their work life.

For persons with SCI, an important facilitator for staying employed was the possibility to hire an assistant helping them with self-care and health management tasks, with preparing themselves for going to work and with transportation. Although this solution was strongly appreciated by persons without family or partner support, they experience challenges regarding the financing of such assistance services by the insurances.

The assistance contribution is also not calculated in right way. For example, when I was pregnant, I couldn't pull my pants up well. This is actually an assistance act. But insurance says it's a 1-min assist so I can't make an employment contract for more than this 1 min. Of course, my assistant won't travel from here to the other city where I work to pull up my pants for a minute after catheterization. That's where it fails. In such completely, completely idiotic solutions. There is again the personal involvement of my colleague, who is pulling my pants up here (E_SCI_1).

Some participants with ABI reported that they continuously struggle to prove that their work-related difficulties result from their injury, which is the basis that they get financially compensated by the insurance. They also complained that some services and health-related aids which were indispensable to stay employed were not paid by the insurance. All these aspects led to a deep frustration and distrust toward insurance companies.

You always have to prove that it's no longer possible. I have to prove that I have a disability, that something still remains. Now of course they stopped the payments again because I might be able to work more. However, this is not permanent. All this stuff is a stumbling block. And that is also a hindrance to keep the job. Because that takes so much strength, so much energy. It's again, and it's an infinite spinning circle. You really feel let down (ABI_6).

#### Systems and Policies

##### Societal Attitudes

Both groups of participants complained about a lack of awareness regarding the consequences of their disability in the society resulting in negative attitudes and prejudices at the workplace or when searching support by the IV. Participants expressed their wish for a more inclusive and tolerant society that would be aware of the needs of people experiencing health-related limitations.

People with SCI also felt discriminated during job interviews and with regard to internal promotions due to their limited mobility and their limited ability to work full-time. However, sometimes they were not feeling able to fulfill the requirements of such position because of their SCI-related limitations.

##### Labor Market

The dynamic development of modern labor market was experienced both as barrier and facilitator for sustainable employment. While flexible work schedules and the possibility for home office resulting from digitalization in the labor market was perceived beneficial for both condition groups, the automatization and computerization of simple tasks was experienced as particularly threatening for people with ABI. For both groups, the high competitiveness and profit orientation in the labor market and the missing consideration of workers' well-being were perceived a major barrier for sustainable employment. Such attitude burdens disabled employees with a high pressure to perform, which threatens disability management and work-life balance. In particular, participants with ABI perceived that there is no place for them anymore in such a labor market and reported that they feel less valuable for today's society because they have supposedly not much to offer in a competitive work environment. At the same time, some participants also expressed that they think they can contribute by doing what they are good at but simply with a slower pace and that they can teach others how to be more patient and tolerant.

It's just competition, it's not collaborative thinking. This is something very bad in my understanding. I struggle with this world. In a framework where money is at stake and the goal is to make the most profit, people with a disability naturally have no place. Because money has much more value than human (ABI_6).

##### Disability and Social Security Policies

Policy regulations were reported both as barriers as well as facilitators by people with SCI as well as ABI. For example, limited long-term support after integration for persons with 75% or higher disability benefit caused frustration, disappointment and sense of abandonment among persons who despite having a full disability pension felt able to work a small amount of time.

While participants with SCI valued the policy regulation that public spaces and buildings need to be accessible for people with mobility limitations, they perceived social security policy regulations regarding the maximum income one can receive to be eligible for disability benefits as hampering for a sustainable career development. This barrier was not mentioned by people with ABI who generally experience limited career opportunities due to their condition-specific limitations.

Among people with ABI, even participants who were employed at the time of our study feared that in case they would become unemployed, finding a new job would be very difficult because of the incompatibility of their limitations with the competitive labor market. Concurrently, they were also aware that their disability benefits are not sufficiently high to maintain their former standard of living, increasing their economic insecurity.

At the moment I am one of the people who they are killing. If my family didn't help me and it wouldn't work with my own studio, then—well, the social welfare finished last year. And I still don't get an IV, because the IV doesn't understand why I functioned normally 10 years after the incident and why it doesn't work anymore. They don't acknowledge the connection (ABI_2).

### Prioritization of Factors

In the second part of the focus groups and the interviews, participants were asked to pick the three most supporting and the three most hindering factors for staying employed. The majority of the participants, independently of their health condition, pointed toward the importance of the work organization, the ability to self-advocate and communicate as well as the key role of a supportive social environment, both at work and in private life.

For me the goodwill of others is crucial. I am working now with a young team of architects having office in an old postindustrial building. I call them when I am in a front of the door and they lift me up through stairs. They hired us and it is absolutely no problem for them to help a wheelchair user. I mean this goodwill. Or when I'm working on construction sites, you have to tell the builder very clearly: “MR. X is coming in a wheelchair, is that a problem for you?” Over the years, I've practically never had a negative experience when someone would say: “Oh, no, I'd rather not have that.” I've experiences really a lot of goodwill over the last 35–3 years. (SCI_7)

People with SCI particularly mentioned the facilitating role of a good self-management of one's health to prevent secondary health conditions, while people with ABI highlighted the importance of being truly understood, which they experienced by peers and in support groups from Fragile.

Understanding and not pity for the situation. Yes, that is important in the job. Because pity always pulled me down. On the other hand, it is extremely important to understand that certain things may not work, or not so well, or whatever (ABI_7).

Most of the key barriers for sustainable employment mirrored the key facilitators. Both people with SCI as well as ABI indicated as key barriers a poorly accessible working place, inflexible working conditions and the lack of ability to communicate. Participants with SCI highlighted the negative impact of a frequently changing health status accompanied by secondary health conditions, while participants with ABI emphasized the fluctuating performance and fatigue. Both groups pointed to the negative experiences when looking for support by the IV or the health insurance.

So if you can't defend yourself, you will be run over. Unjustified. For 100%. I've already been to federal court. I look at every decision. I learned how to argue. But that is something that you have to learn and be able to do (SCI_7).

For people with ABI, difficulties in navigating through administrative procedures of the social security system were particularly distressing due to their limited attention and concentration span. Another key barrier was the invisibility of their ABI-related impairments and a poor awareness of others regarding their limitations at work and in daily life.

As the most important factor to improve their work situation, people with SCI indicated a stable health status, a well-adjusted work place and more customized support by the social security system.

I would change the whole social security system. Because you have to fight with the IV, otherwise you have to fight with the Suva. And everyone judges you differently, that's chaos. Also, the IV is just so narrow-minded. They just say, wheelchair users, that's it. But what comes with this: the incontinence, the care, the surgery, they don't see that. Just a wheelchair (SCI_3).

Persons with ABI expressed a need for a qualified support person who is knowledgeable about the procedures of the health and social security system as well as a wish for a more inclusive and tolerant society.

The cantonal spirit in this country—the historically grown 11 social insurances. And every doctor, every service provider, every insurer says, “My little garden, my little garden.” And those who somehow try to think together are extremely rare. That is why it is so important that these studies are done. That one can draw the conclusions about the famous synergies that are always talked about. That just a little bit of understanding grows. Not only for brain injuries, but also for us humans (ABI_2).

## Discussion

The present study identified facilitators and barriers to sustainable employment from the perspective of persons with SCI and ABI. Identified factors pertained to the three biopsychosocial areas of personal, impairment-related and environmental factors. Similarities between persons with SCI and ABI typically appeared at the level of personal and environmental factors (e.g., self-management, need of long-term support services), while impairment-related issues (e.g., secondary health complications) differed between the two conditions. A high potential to support sustainable work became apparent at the level of environmental factors, particularly in long-term support, ongoing sensitization at the workplace and customizing disability policies. Additional areas for interventions may include psychological support with the aim to empower individuals (e.g., self-awareness, self-advocacy or self-management training) and medical or therapeutic interventions targeting the interaction between work and secondary health issues.

The factors identified in our study are in line with those that were found to be associated with sustaining work in the long term in the international literature (6, 7, 30). They also overlap with key determinants of a successful RTW (31–37), indicating that some of the factors that are important for RTW remain significant in the long run. For example, being understood and supported by others at work and in private life is important for both RTW and sustaining employment as found by previous research (6, 7). However, as shown in our study and previously reported, support at the workplace tends to diminish over time^1^. Our findings further confirm disability-related discrimination practices experienced by employees (38) and a higher risk of job loss due to the accumulation of sickness absences (39). Promoting understanding and awareness of the disease and its work-related consequences among employers, colleagues, and those affected, appears to be a key intervention target to support sustainable work. Our results also showed that learning how to live with a disability is a crucial and ongoing process for staying employed. The person has to acquire specific work, personal and disability-related skills which need to be complemented with an adjusted and supportive work environment. This emphasizes the importance of a good person-job match (40). A common reflection by our participants was that such factors are likely to be similar for non-disabled persons, but that their impact on individual's work ability and work situation is most often disability-specific. For example, it is complicated for a single parent to organize work but it is even more challenging for a single parent with SCI to do this due to less flexibility and additional time needs for health management, support organization and commuting.

Our study also highlights the importance of a holistic approach to sustainable employment for persons with a disability, taking account of personal, injury-related and environment factors as well as the interdependency between these areas (41). For instance, the severity of an impairment defines the person's need for adjustments and support from the environment as well as the influence and importance of personal factors such as a good self-management. Overall, environmental factors appear to be the most crucial areas for improving sustainable work. At the micro level, the work and social environment as well as support services over the life course appeared as critical, at the meso level the disability insurers and at the macro level social security and labor market policies.

Facilitators and barriers identified by individuals with SCI and ABI turned out to be largely similar. Key facilitators included adapted work tasks, well-accessible workplaces with supportive employer and colleagues. The labor market dynamics and policies were perceived as challenging for both groups. Persons with ABI felt that fulfilling the requirements of the highly competitive and fast changing labor market, including the need for further education, was not achievable for them due to their cognitive limitations. People with SCI emphasize that there are not enough part-time jobs and that interesting positions often require a high level of commitment, flexibility at work, and constant accessibility, which does not meet their health routine needs. Our findings are in line with previous studies emphasizing the need for a more flexible labor market (41) to be inclusive for persons with diverse ability profiles, because it is extremely difficult to merge disability self-management with functioning in the competitive labor market^3^ and prioritization of work performance over health management proves unsustainable in the long term.

Having a professional support and guidance was experienced as crucial or missed dearly during the RTW phase and as critical in the long-term by those affected, their employers^1^ and health professionals^2^. Employers and employees with positive RTW experiences often express that they miss such long-term support, when problems at work arose for example due to changes in the work environment, a restructuring of the company or a new supervisor or colleague. It is thus crucial that long-term support services (42–45) are offered, accessible and financed for all persons with a disability especially since current policies do not account for the changing, temporal impact of disability on activities of daily life and potential fluctuations in work ability (41).

Currently, a wide range of different health and work-related services are offered in Switzerland. However, these services are fragmented and their availability depends on the region and the policy of the individual provider. Such a system leads to a multitude of interfaces between the different settings, such as rehabilitation, vocational integration, work retention and prevention services. Therefore, a bridging effort involving the individual professional, the service provider and the social security and policy regulations (micro, meso, macro level) is needed to improve the current situation. Decreasing fragmentation and creating coordinated services following people from acute care to rehabilitation and long-term work retention with dropout prevention would close the gap between stakeholders and might improve effectiveness and efficiency of the current support structure.

Differences between the two conditions became mainly evident at the level of impairment-related factors. However, the impact of the respective health condition on work performance was comparable, including extra-time needs for completing work tasks, resting after the working day and self-management of the disability and its consequences. Due to the dynamic nature of these chronic conditions, self-management develops over time^3^ and requires complex personal skills like self-advocacy and good communication with the workplace, health and insurance professionals and at home. For example, if a person needs to leave work in the middle of the day to go for a therapy or is unavailable for meetings in the morning because needs more time to get ready to work self-advocacy in explaining why self-management is important becomes crucial. To maintain good health, it is important to integrate medical management into daily routines (46–48). Better self-management of chronic condition was related to improved biological parameters, fewer symptoms (49, 50) and better daily role management (51, 52).

Differences between SCI and ABI also related to the visibility of the impairment. The invisibility of injury-related impairments and the difficulty to directly quantify their impact on work ability reflects a particular challenge for the ABI group, triggering further problems such as not being understood or falsely perceived as lazy at work. This issue has been further investigated by Teindl et al. (53) who reported that having an invisible disability makes keeping a job more difficult. Persons with SCI reported that although their disability is visible to others, which may create a barrier when looking for new jobs, their secondary health conditions are not, which often leads to underestimation of the difficulties they are dealing with.

Differences at the level of environmental factors are perceived with regard to the health and social security services available. Although the Swiss health care and social security system provides good treatment over the years with medical and vocational rehabilitation, the complexity and organization of services puts a burden on the injured individuals as shown especially by ABI case. While both groups reported barriers related to the health care system, participants with SCI strongly highlighted how helpful the support network and coordination of services and settings provided by specialized centers and patient organizations was for their work reintegration and for their further work-life. The Swiss Paraplegic Group provides a comprehensive support for people with SCI including inpatient and outpatient medical, psychological, social and vocational rehabilitation and integration services along individuals' life course. After discharge from their initial inpatient rehabilitation, individuals may seek further support from ParaHelp (i.e. a specialized home care institution for persons with SCI) or from the Swiss Paraplegic Association (SPA) that provides life and peer counseling and helps with housing, legal and financial issues. Additional facilitators specific for the Swiss system include the disability benefits, reduced weekly workload and the possibility to reassess the work ability in case of health deterioration. Furthermore, support for employers during the reintegration process and the IV covering the costs of the workplace accommodations may reduce employers' negative attitudes toward employees with a disability (39, 54). Altogether, the SCI example shows that having an integrated pathway from acute to long-term care considering personal, injury-related and environmental factors facilitates labor market participation of people with a disability.

### Strengths and Limitations

A main strength of our study is that we collected a broad range of data from 14 focus groups and four interviews with persons with SCI or ABI. With the interviews we were able to include the valuable experience of severely injured people who would not have been able to participate in the focus groups. The inclusion of people with two different health conditions is another strength of our study. While combining two seemingly different health conditions like SCI and ABI might be seen as a challenge (i.e., due to peculiarities missed), we think that this allowed us to comprehensively capture facilitators and barriers to sustainable work for persons with neurological conditions. SCI and ABI show a large overlap, while a part of the ABI population tends to encounter mobility limitations due to the injury and over 39% of people with SCI sustain also an ABI (15). Together these two health conditions represent most of the challenges experienced by other neurologic conditions and as shown in our results partly overlap and partly complement each other.

However, our study has also some limitations. First, the Swiss labor market and social security system differs significantly from other countries. Our results, especially the ones on environmental factors, are therefore not generalizable to countries with different labor market structures and social security systems. Moreover, there is a risk of a positive selection bias in our study because the majority of our participants managed to stay employed in the long run. Despite we formulated the study announcement openly and emphasized that we are also interested in people who are currently not working, only a few participants who are unemployed were recruited. Additional research focusing on the experiences of people with a disability who left the labor market should be conducted to extend our findings.

### Practical, Policy, and Research Implications

In our study, we identified barriers and facilitators for sustainable employment that were considered critical by persons with SCI and ABI. Among the identified biopsychosocial factors, the modifiable ones could be addressed by the following interventions:

- communication skills, which are crucial to self-advocate and to inform about the work-related consequences of the disability, should be promoted through trainings offered by vocational integration specialists or psychologists during and after initial rehabilitation,
- medical and therapeutic interventions could improve the individuals' work-related functioning (44),
- long-term support services for people with a disability with an easy access to vocational professionals and psychological support should be established,
- vocational support services for people with low weekly workloads should be provided as a measure of social integration to include people who are willing to work in the labor market,
- a coordinated insurance support should be offered to those affected and their employers who also considered cooperation with disability insurance as stressful and inefficient^1^,
- the disability pension levels should be easier to adapt to allow for an adequate career development without having to fear an income loss due to a disability pension reduction,
- more flexible labor market policies accounting for changing work related abilities of persons with a disability should be created,
- awareness campaigns should be initiated with regard to what it means to live and work with a particular disability to create a more inclusive society.

Due to interplay of biopsychosocial factors the intervention in one area will have a positive consequence in the other one, for example improving self-management or creating of healthy working conditions should lead to less health problems.

Thanks to exploring experiences of participants with SCI and ABI, we identified similarities in their perspectives, that could be further explored as basis to formulate guidelines to support sustainable employment for neurological conditions in general or to shed light on the needs of an aging population who mainly deal with limitations in mobility and cognitive functioning.

Due to changes in functioning related consequences of the injury over time (55, 56), future research should employ a life course approach and involve the perspective of the key stakeholders. Mixed-methods research would be particularly valuable to assess effectiveness of strategies and services. By combining biographical interviews with time-updated longitudinal data on labor market participation and its biopsychosocial predictors, guidelines on how to support sustainable employment of persons with a disability could be developed. Our study is a first qualitative step of this process taking a long-term view on the employment of persons with SCI or ABI and coming up with factors affecting their sustainable work.

## Conclusion

We identified a variety of strongly interrelated factors at the level of the person, the person's impairment and the environment that facilitates or hinders sustainable employment of persons with SCI or ABI. Identified barriers and facilitators should be addressed during first RTW. In addition, an easily accessible professional support should be granted throughout the work-life of the injured worker, if needed. Interventions may concern the level of the person (e.g., self-management, self-advocacy and communication training), medical-therapeutic interventions (e.g., work endurance training) and the environment, including the work environment (e.g., creation of health/sustainable working conditions; awareness raising at employer/ co-worker). In addition, changes in the service structure (e.g., long-term support services across settings of rehab—integration—prevention) and on the policy level (e.g., adapting of disability pension scheme) should be further evaluated and addressed. Ensuring a good match between person's abilities, interests and needs with the job's demands and work environment under consideration of external biopsychosocial influences plays a crucial rule in all interventions that aim to ensure sustainable work.

## Data Availability Statement

The datasets presented in this article are not readily available in order to protect the anonymity of participants. Requests to access the datasets should be directed to KK, katarzyna.karcz@paraplegie.ch.

## Ethics Statement

The studies involving human participants were reviewed and approved by the Ethical Committee of North-West and Central Switzerland (EKNZ, study reference 2018-01317). The patients/participants provided their written informed consent to participate in this study.

## Author Contributions

MF and KK were responsible for designing the conceptual framework of the study. MF and BS recruited participants. MF, KK, and BS conducted focus groups and interviews. KK coded and together with MF. BS and US analyzed the data and prepared the paper. SS provided valuable input from vocational rehabilitation practice. All authors contributed to the article and approved the submitted version.

## Funding

This work was supported by the Swiss National Science Foundation under Grant No. 10531C_173322/1.

## Conflict of Interest

The authors declare that the research was conducted in the absence of any commercial or financial relationships that could be construed as a potential conflict of interest.

## Publisher's Note

All claims expressed in this article are solely those of the authors and do not necessarily represent those of their affiliated organizations, or those of the publisher, the editors and the reviewers. Any product that may be evaluated in this article, or claim that may be made by its manufacturer, is not guaranteed or endorsed by the publisher.

## Footnotes

^1^Schiffmann B, Finger ME, Karcz K, Staubli S, Trezzini B. Factors related to sustainable employment of people with acquired brain injury or spinal cord injury: the employer's perspective. *Front Rehabil Sci*.

^2^Finger ME, Schiffmann B, Karcz K, Staubli S, Escorpizo R. Factors influencing sustainable employment of persons with acquired brain injury (ABI) or spinal cord injury (SCI): A qualitative study evaluating the perspective of health and work professionals. *Front Rehabil Sci*.

^3^Qama E, Rubinelli S, Diviani N. Factors challenging the integration of chronic illness self-management: a scoping review of qualitative data. *Plos One (under review)*. (2022).
